# Activation of brown adipose tissue by a low-protein diet ameliorates hyperglycemia in a diabetic lipodystrophy mouse model

**DOI:** 10.21203/rs.3.rs-2701883/v1

**Published:** 2023-03-31

**Authors:** Marcos Munoz, Alexa Zamudio, Maximilian McCann, Victoria Gil, Pingwen Xu, Chong Wee Liew

**Affiliations:** University of Illinois at Chicago; University of Illinois at Chicago; University of Illinois at Chicago; University of Illinois at Chicago; University of Illinois at Chicago; University of Illinois at Chicago

**Keywords:** Low-protein diet, lipodystrophy, hyperglycemia, brown adipose tissue

## Abstract

Long-term ad libitum dietary restrictions, such as low-protein diets (LPDs), improve metabolic health and extend the life span of mice and humans. However, most studies conducted thus far have focused on the preventive effects of LPDs on metabolic syndromes. To test the therapeutic potential of LPD, we treated a lipodystrophy mouse model IR^FKO^ (adipose-specific insulin receptor knockout) in this study. We have previously shown that IR^FKO^ mice have profound insulin resistance, hyperglycemia, and whitenng of interscapular brown adipose tissue (BAT), closely mimicking the phenotypes in lipoatrophic diabetic patients. Here, we demonstrate that 14-day of LPD (5.1% kcal from protein) feeding is sufficient to reduce postprandial blood glucose, improve insulin resistance, and normalize glucose tolerance in the IR^FKO^ mice. This profound metabolic improvement is associated with BAT activation and increase in whole body energy expenditure. To confirm, we showed that surgical denervation of BAT attenuated the beneficial metabolic effects of LPD feeding in IR^FKO^ mice, including the ‘browning’ effects on BAT and the glucose-ameliorating results. However, BAT denervation failed to affect the body weight-lowering effects of LPD. Together, our results imply a therapeutic potential to use LPD for the treatment of lipoatrophic diabetes.

## Introduction

Metabolic syndrome, a worldwide epidemic with high socioeconomic cost, is a complex disorder driven primarily by obesity^1^. The key features of metabolic syndrome include hyperglycemia, dyslipidemia, insulin resistance, non-alcoholic fatty liver, and many other associated metabolic abnormalities^2^. Great strides have been made toward managing the metabolic profiles of metabolic disease patients, but effective strategies for fixing metabolic syndrome still need to be discovered.

Dietary intervention to prevent or control obesity and type 2 diabetes has been used for decades and can be highly effective and affordable. Long-term ad libitum dietary restriction (i.e., 20–40% reduction in food intake) has been shown to improve metabolic health and extend the life spans of mice and possibly humans^3–6^. Since long-term voluntary dietary/caloric restriction is impractical for most people, diets that alter the levels of specific macronutrients without decreasing caloric consumption are seen as more sustainable by researchers and the public^7^. For decades, research has focused on the relationship between dietary carbohydrate/fat ratio and the homeostasis of body weight and blood glucose control^8,9^. The role of dietary protein in metabolism has begun to be appreciated only recently^5,10−14^.

Recent comprehensive macronutrient analyses have demonstrated that a low-protein (LP), high-carbohydrate diet is as potent as the caloric restriction in preventing aging-induced metabolic dysfunctions in mice^5,15^. Consistently, LPD has been shown to promote weight loss, enhance insulin sensitivity, and increase energy expenditure in wild-type rodents^16,17^. In addition to data from model organisms, multiple long-term prospective cohort studies have shown that LPDs are associated with decreased mortality and cancer in humans. Conversely, high-protein diets are associated with insulin resistance, diabetes, and mortality^6,18−20^. Consistent with these prevention studies, a recent randomized, controlled trial showed that dietary protein restriction (0.8 g of protein/kg body weight) improved metabolic dysfunction in patients with metabolic syndrome^21^, further suggesting a therapeutic potential of LPD. However, the exact beneficial metabolic effects and mechanisms of LPD on diabetes, especially lipodystrophy-associated diabetes, are still unknown.

In this work, we used an adipose-tissue specific insulin receptor knockout (IR^FKO^) mouse model that we have recently developed^22^ to mimic the phenotypes observed in lipodystrophy diabetes patients, i.e., hyperglycemia, insulin resistance, glucose tolerance, extremely low body fat, and whitening interscapular brown adipose tissue (BAT). We found that two weeks of LPD feeding profoundly decreased body weight, enhanced BAT browning, reduced postprandial blood glucose, improved insulin resistance, and normalized glucose tolerance in IR^FKO^ mice. Mechanistically, we showed that LPD-induced glucose normalization but not body weight reduction is BAT activation-dependent. Our results imply a therapeutic potential to use LPD for the treatment of severe lipoatrophic diabetes.

## Results

### A low-protein diet improves glucose homeostasis in lipodystrophic diabetic mice.

**Figure 1:**
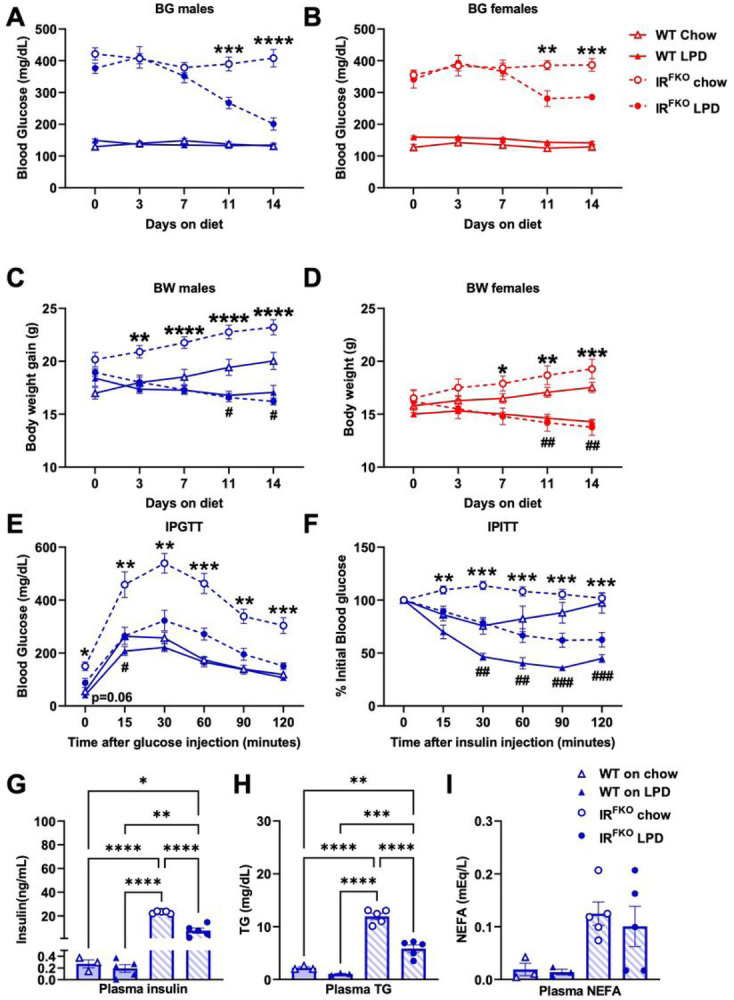
A low-protein diet improves glucose homeostasis in lipodystrophic diabetic mice. Blood glucose (A)(B) and body weight (C)(D) of IR^FKO^ (circles) and WT (triangles) mice fed ad libitum chow diet (empty lines) or low protein diet (solid lines) in males (blue) and females (red) were measured at fed conditions every 3 days for 2 weeks after the initiation of dietary treatment. Intraperitoneal glucose tolerance (E) and insulin tolerance (F) tests for male WT and IRKFO mice fed for 2 weeks with LPD or chow diet. Plasma insulin (G), triglycerides (H) and non-esterified fatty acids (J) of male IR^FKO^ and WT mice. N=5–8 per group. All data are presented as the mean ± standard error of mean (SEM) and were analyzed by two-way ANOVA test (Figures A-F) or one-way ANOVA test (G-I) with a threshold for significance of 0.05 adjusted P value. *P>0.05; **P>0.01; ***P> 0.001; ****P>0.0001.

To determine the therapeutic potential of the LPD, we subjected both male and female WT (IR^flox/flox^) and IR^FKO^ (IR^flox/flox^, AdipoQ-Cre) lipodystrophic diabetic mice to either a chow or LP diet. We monitored body weight and blood glucose twice a week. Strikingly, we observed that the fed postprandial blood glucose of the male IR^FKO^ mice started to improve one week after the protein restriction and became close to normoglycemic at the end of the two-week treatment compared to those on a chow diet (Fig. 1A). We consistently observed a similar LPD-induced glucose-ameliorating effect in the female IR^FKO^ mice (Fig. 1B). In contrast to the beneficial effect observed in the lipodystrophic diabetic mice, we did not detect significant changes in the postprandial blood glucose in both the male and female WT mice after LPD, which is, however, likely due to the lean and healthy nature of the control mice (Figs. 1A & 1B).

In consistency with previous studies demonstrating body weight loss after LPD treatment^5,11,23,24^, male and female IR^FKO^ mice lost about 14% of their body weight at the end of the two-week LPD treatment (Figs. 1C & 1D). Reduction in body weight could potentially lower blood glucose, improve insulin sensitivity, and ameliorate metabolic health of the IR^FKO^ mice, however, the lower body weight observed in the IR^FKO^ mice on LPD is unlikely to be the major contributing factor for the BG normalization, as the WT mice on the LPD group also lost about 7% of their body weight (Figs. 1C & 1D) without noticeable changes in the postprandial blood glucose levels (Figs. 1A & 1B). Blood glucose-lowering observed in IR^FKO^ after LPD treatment is likely contributed by improved glucose tolerance and insulin sensitivity, as shown by the glucose and insulin tolerance tests (Figs, 1E & 1F). Due to the ectopic lipid accumulation, the IR^FKO^ mice are severely hyperinsulinemic and hyperlipidemic. Interestingly, after the acute short-term protein restriction, in addition to the blood glucose-lowering effect, LPD IR^FKO^ mice also have significantly reduced plasma insulin and triglyceride (TG) (Figs. 1G & 1H). Notably, despite reducing TG in the IR^FKO^ mice, LPD did not affect plasma non-esterified fatty acids (NEFA) levels in WT and IR^FKO^ mice (Fig. 1I).Taken together, our data showed that acute short-term protein restriction appears to have beneficial metabolic effects even in the severely diabetic lipodystrophic mice model.

### LPD activates BAT glucose uptake and thermogenesis.

**Figure 2:**
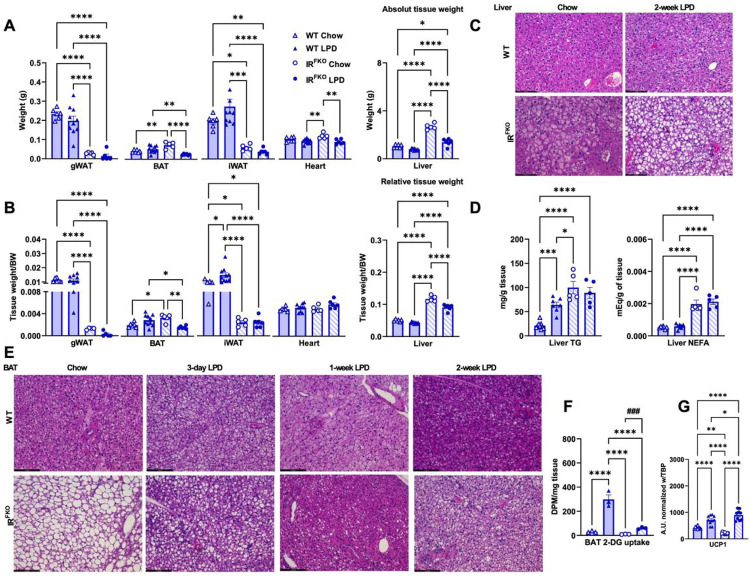
LPD activates BAT glucose uptake and thermogenesis. Absolute (A) and relative (B) tissue weights for male IR^FKO^ and WT mice fed for 2 weeks with chow diet (empty lines) or LPD (solid lines). (C) Representative images of H&E-stained sections of liver from male IR^FKO^ and WT mice fed for 2 weeks with chow diet or LPD. Scale bar, 50 μm. (D) Liver triglycerides (TG) and non-esterified fatty acid (NEFA) content for male IR^FKO^ and WT mice fed for 2 weeks with chow diet (empty lines) or LPD (solid lines). (E) Representative images of H&E-stained sections of BAT from male IR^FKO^ and WT mice fed for 3, 7 and 14 days with chow diet or LPD. Scale bar, 100 um. (F) BAT 2-DG glucose uptake for male IR^FKO^ and WT mice fed for 2 weeks with chow diet (empty lines) or LPD (solid lines). Values are expressed as DPM per mg of tissue. (G) Ucp1 mRNA levels in BAT of male IR^FKO^ and WT mice fed for 2 weeks with chow diet (empty lines) or LPD (solid lines). Values are normalized to TBP gene levels. N=5–8 per group for A-E and G. N=3 per group for F. All data are presented as the mean ± standard error of mean (SEM) and were analyzed by one-way ANOVA test with a threshold for significance of 0.05 adjusted P value except in (F) were comparison between IR^FKO^ chow vs IR^FKO^ LPD was performed on separate two-tail student t-test. *P>0.05; **P>0.01; ***P> 0.001; ****P>0.0001;###P>0.001

To determine potential contributing factors for blood glucose and body weight reduction in the IR^FKO^ mice after LPD, we euthanized the mice after the dietary treatment and collected tissue samples. As reported previously^22^, loss of white adipose tissue caused organomegaly in the IR^FKO^ mice, as shown by the significantly increased BAT and liver tissue weight (Fig. 2A). In line with the body weight data, we observed that LPD treatment significantly reduced BAT, heart, and liver tissue weight in the IR^FKO^ mice (Fig 2A). Since LPD treatment has a strong body weight reduction effect in the IR^FKO^, reduction in the tissue weight could be directionally proportional to the body weight reduction. To confirm, we also determined the body weight-normalized tissue weight. Our data showed that the relative liver and BAT tissue weight in the IR^FKO^ mice after LPD are indeed lowered (Fig. 2B).

Short-term LPD has previously been shown to promote lipid accumulation in the liver of WT rat^25^. Consistently, our H&E staining showed larger, lipid-containing vacuoles in the IR^FKO^ liver after LPD treatment (Fig. 2C) despite a reduction in the liver tissue weight after LPD (Figs. 2A & 2B). To confirm whether the protein restriction treatment would exacerbate IR^FKO^ mice hepatosteatosis, we extracted lipids from the liver of IR^FKO^ mice before and after LPD treatment. Consistently with previous study^25^ and histological data, we observed a significant increase of TG in the WT liver after LPD feeding (Fig. 2D). In contrast, unexpectedly, our data showed that LPD did not increase liver TG and NEFA in the IR^FKO^ mice (Fig. 2D), which is inconsistent with the larger vacuole observed histologically. The reason for this discrepancy requires further studies for clarification.

In contrast, H&E staining showed dramatically reduced lipid droplet size in IR^FKO^ brown adipocytes 2 weeks after LPD treatment (Fig. 2E). Reduction in the lipid storage in IR^FKO^ BAT occurred as early as 3 days after the onset of LPD treatment, and most lipid storage was depleted after one week of protein restriction (Fig. 2E). We know that excessive lipid accumulation in brown adipocytes causes BAT whitening and downregulation of BAT metabolic functions, as seen in the IR^FKO^ mice^22^. To investigate whether reducing lipid storage in brown adipocytes restores IR^FKO^ BAT function, we first examine BAT *in vivo* glucose uptake capability. Our in vivo 2-deoxy-glucose uptake assay showed that LPD diet significantly increased glucose uptake in the WT BAT and fully restored glucose uptake capacity in the IR^FKO^ mice BAT, which was initially impaired by the excessive lipid accumulation (Fig. 2F). In addition, we also examine thermogenic markers’ gene expression. Our results showed that LPD feeding activated and fully restored downregulated UCP1 expression in the IR^FKO^ mice (Fig. 2G). Taken together, our data clearly demonstrated that LPD is a potent activator of BAT metabolic functions.

### LPD increases energy expenditure in both WT and IR^FKO^ mice.

**Figure 3:**
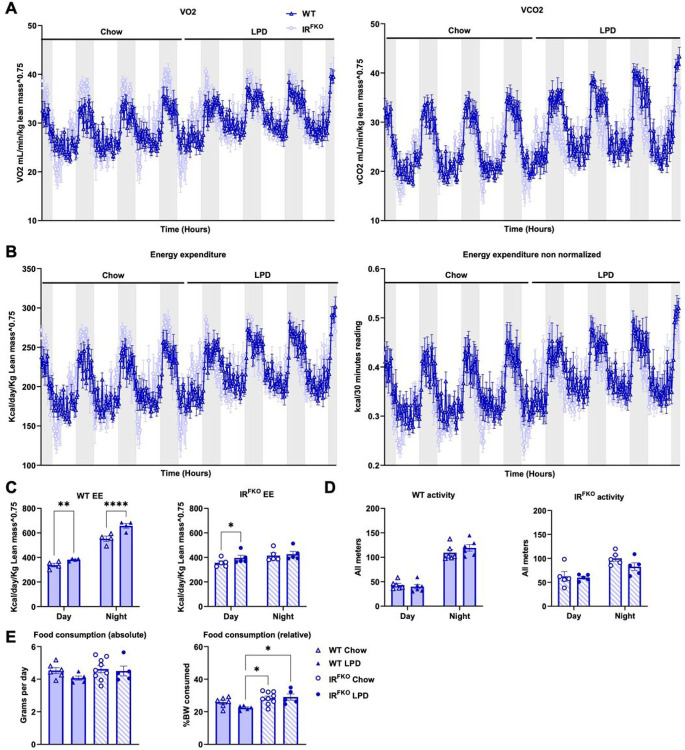
LPD increases energy expenditure in both WT and IR^FKO^ mice. (A) Oxygen consumption (VO2, left panel) and carbon dioxide production (VCO2, right panel) analyzed by indirect calorimetry for 3 days in chow then 3 days in LPD diets in male WT (dark blue lines) and IR^FKO^ (light blue lines) mice. (B) Normalized (left panel) and non-normalized (right panel) energy expenditure.Values are expressed as kcal/day/g lean masŝ0.75 for normalized panel and as kcal/30 minutes for non-normalized panel. (C) Normalized energy expenditure averages for day and night periods of WT (left panel) and IR^FKO^ (right panel). (D) Spontaneous physical activity measured by x and y axis beam breaks. Values are displayed as measurements for all meters in arbitrary units. (E) Absolute (left panel) and relative (right panel) food consumption for chow (empty circles) and LPD diet (solid circles) periods. Values are expressed as average grams consumed by day for absolute values and % of BW consumed per day for relative values. N=5–8 as per group. All data are presented as the mean ± standard error of mean (SEM). Energy expenditure in figure (C) was analyzed using paired two-tailed student’s T test comparing the average of day/night period values for each mouse before and after diet switch. Food consumption measurements in figure E were analyzed by one-way ANOVA test with a threshold for significance of 0.05 adjusted P value. *P>0.05; **P>0.01.

To determine how the ablation of the insulin receptor in adipose tissues and consumption of LPD modulate energy balance in mice, we subjected both the WT and IR^FKO^ mice to metabolic measurements in metabolic cages (Promethion Metabolic System, Sable). Mice were started on a chow diet and then switched to LPD after 5 days of measurement. Interestingly, we observed that IR^FKO^ mice consistently showed lower oxygen consumption (VO2), carbon dioxide production (VCO2), and energy expenditure (EE) at the onset of the light cycle compared to WT mice on a chow diet (Figs. 3A & 3B). These differences were completely abolished by LPD feeding (Figs. 3A & 3B). In line with previous studies^16,26^, we also observed that a LPD sustainably upregulates VO2, VCO2, and EE in the WT mice (Figs. 3A & 3B). Consistent with the changes observed in the BAT, IR^FKO^ mice also showed significant upregulation of EE after the LPD feeding. However, unlike previous reports on a progressive increase in EE induced by LPD^27–29^, we observed an extremely rapid (within 12 h) increase in EE in both the WT and IR^FKO^ mice after the diet switch. Even though the LPD affected both the light and dark cycles, the LPD-induced EE effect seems more pronounced for the light cycle, especially in the IR^FKO^ mice. Using the hourly data, we performed a paired-wise comparison analysis. Our data showed that LPD induces WT mice EE in both the light and dark cycles but only in the light cycle in the IR^FKO^ mice (Fig. 3C). In addition, we also observed that increases in EE after LPD feeding were not attributed to changes in physical activity (Fig. 3D). In addition, unlike previous reports on LPD-induced hyperphagia^16,30,31^, we did not observe an increase in food consumption in the WT mice on LPD (Fig. 3E). Despite significantly lower body weight at the end of the LPD treatment (Figs. 1B & 1C), IR^FKO^ mice tend to consume a similar amount of food before and after the LPD switch. Our data suggests that the reduction in body weight after LPD treatment observed in both WT and IR^FKO^ mice is likely attributed to an increase in EE but not food consumption.

### BAT denervation abolished the LPD-induced blood glucose-lowering effect.

**Figure 4:**
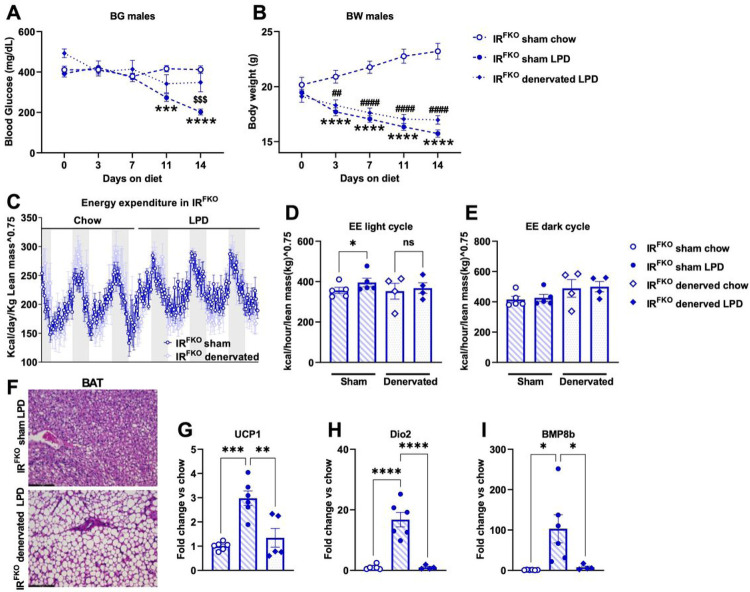
BAT denervation abolished the LPD-induced blood glucose-lowering effect. BAT surgical denervation performed in IR^FKO^ mice. In short, all five branches of intercostal sympathetic nerves connecting to the right and left BAT fat pads were identified, carefully isolated, and sectioned. Sham mice underwent the same procedure without sectioning intercostal sympathetic nerves. Then, mice were subjected to 14 days of low protein or chow. Blood glucose (A) and body weight (B) of chow fed sham IR^FKO^ (empty circles), LPD fed sham IR^FKO^ (solid circles) and LPD fed denervated IR^FKO^ mice (solid rhomboids). (C) Normalized energy expenditure of sham (dark blue line) and denervated IR^FKO^ mice (light blue line) fed ad-libitum chow diet for 3 days before switch to LPD diet for 3 days. (D) Normalized energy expenditure averages for light and dark cycles of sham (circles) and denervated (rhomboids) mice in chow fed (empty symbols) or LPD fed (solid symbols) mice. (F) Representative images of H&E-stained section of BAT for male sham and denervated IR^FKO^ mice fed with LPD. Scale bar, 100 um. (G-I) UCP1, Dio2 and BMP8b mRNA levels for chow fed sham IR^FKO^ (empty circle), LPD fed sham IR^FKO^ (solid circles), and LPD fed denervated IR^FKO^ (solid rhomboids) mice are expressed as fold change relative to sham mice on chow diet. N= 4–6 per group. All data are presented as the mean ± standard error of mean (SEM) and were analyzed by two-way ANOVA test followed by Post hoc Tukey analysis (A & B), paired two-tailed student T test (D & E) or one-way ANOVA test (G-I) with a threshold for significance of 0.05 adjusted P value. *P>0.05; **P>0.01; ***P> 0.001; ****P>0.0001; ^$ $ $^P>0.001 in IR^FKO^ sham LPD vs IR^FKO^ denervated LPD; ^###^P>0.001 in IR^FKO^ sham chow vs IR^FKO^ denervated LPD.

To determine whether activation of BAT is required for LPD-induced blood glucose normalization, we performed BAT denervation on IR^FKO^ mice. Upon recovery, sham and denervated IR^FKO^ mice were subjected to chow and LPD treatment. Interestingly, we observed that LDP failed to lower the blood glucose of BAT-denervated IR^FKO^ mice (Fig. 4A). At the end of the 14-day treatment, LPD-treated IR^FKO^ mice had significantly lower blood glucose compared to IR^FKO^ mice on a chow diet and denervated IR^FKO^ mice on LPD. However, unlike blood glucose, BAT denervation did not affect LPD-induced body weight reduction (Fig. 4B).

To determine the impact of BAT denervation on LPD-induced increases in energy expenditure, we subjected an additional cohort of sham and denervated IR^FKO^ mice to metabolic profile measurements. Similarly, mice were started on a chow diet and switched to LPD after 5 days of measurement. Our data showed that LPD significantly increases the sham IR^FKO^ mice EE during the light cycle, but this stimulatory effect was abolished in the denervated IR^FKO^ mice (Figs.4C & 4D). Consistent with our previous data (Fig. 3C), LPD did not have a significant impact on IR^FKO^ mice dark cycle EE, and this is not affected by BAT denervation (Fig. 4E).

To visualize the impact of BAT denervation on LPD-induced morphological changes, we collected BAT from sham and denervated IR^FKO^ mice after 2 weeks of LPD feeding. Our histological analysis showed that unlike the sham IR^FKO^ mice that lost most of the ectopically accumulated lipid due to lipodystrophy after LPD treatment (Figs. 2E & 4F), BAT of denervated IR^FKO^ mice remained lipid-filled even after LPD feeding (Fig. 4F).

To determine the effects of BAT denervation on thermogenesis, we examined the mRNA expression of well-characterized thermogenic markers. Consistent with the metabolic and histological data, our gene expression analysis showed that BAT denervation completely abolished LPD-induced UCP1, Dio2, and BMP8a upregulation in IR^FKO^ mice (Figs. 4G–4I).

### Fibroblast Growth Factor 21 (FGF21) potentially mediates BAT-facilitated blood glucose lowering effects of LPD in IR^FKO^ mice.

**Figure 5:**
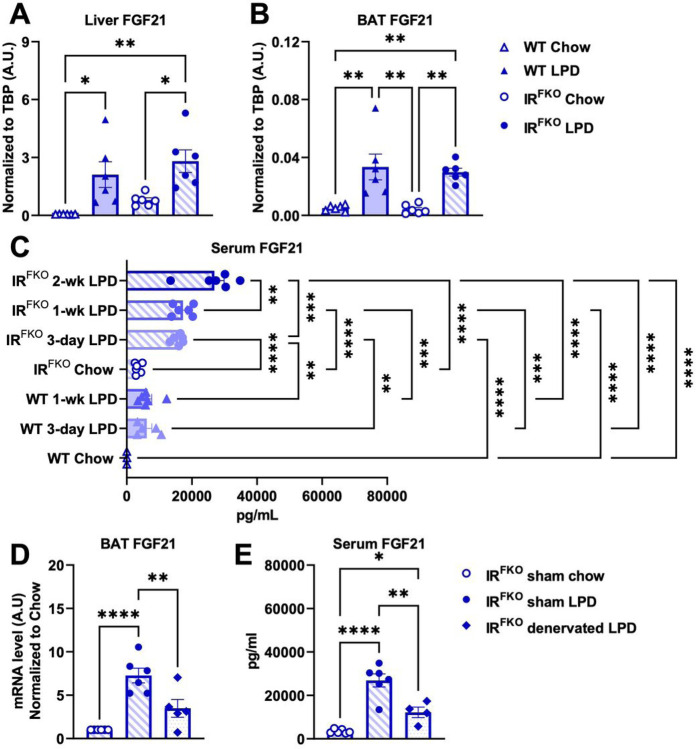
Fibroblast Growth Factor 21 (FGF21) potentially mediates iBAT-facilitated hypoglycemic effects of LPD in IR^FKO^ mice. (A) Liver and (B) BAT FGF21 mRNA levels of male WT and IR^FKO^ mice fed chow (empty symbols) and LPD (solid symbols) for 14 days. Values are expressed as arbitrary units relative to TBP gene expression.(C) FGF21 serum levels for male WT and IR^FKO^ mice fed chow or LPD for 3, 7 and 14 days. (D) BAT FGF21 mRNA levels of chow fed sham IR^FKO^ (empty circles), LPD fed sham IR^FKO^ (solid circles) and LPD fed denervated IR^FKO^ mice (solid rhomboids). Values are expressed as arbitrary units relative to sham mice chow fed. (E) FGF21 serum levels of sham and denervated IR^FKO^ mice fed with chow or LPD diet. N=5–7. All data are presented as the mean ± standard error of mean (SEM) and were analyzed by one-way ANOVA test with a threshold for significance of 0.05 adjusted P value. *P>0.05; **P>0.01; ***P> 0.001; ****P>0.0001.

Previous studies demonstrated that LPD-induced metabolic benefits are at least partially mediated by FGF21^27,28,32^. Since circulating FGF21 is primarily produced by the liver, we first examined FGF21 mRNA expression in the liver. Consistently, we observed a significant increase in FGF21 in WT and IR^FKO^ liver after 2 weeks of LPD feeding (Fig. 5A). Interestingly, we also observed a tendency of increase in liver FGF21 induced by IR^FKO^ under basal condition (Fig. 5A).

Since our data so far supports a crucial role of BAT in mediating LPD-induced blood glucose-lowering effects, we also examined BAT FGF21 expression. Despite extremely low basal expression, we observed a significant increase in the mRNA expression of BAT FGF21 in both the WT and IR^FKO^ mice (Fig. 5B). To confirm that increase in liver and BAT FGF21 expression will indeed increase circulating FGF21 levels, we examined plasma FGF21 in both WT and IR^FKO^ mice at different LPD-treatment time points. Our immunoassay data confirmed that LPD indeed increases circulating FGF21 levels in both the WT and IR^FKO^ mice (Fig. 5C). Intriguingly, LPD-fed IR^FKO^ mice showed significantly higher circulating FGF21 levels compared to the LPD-fed WT mice in all the time points examined (Fig. 5C).

To determine the impact of BAT denervation on LPD-induced FGF21 expression, we examined FGF21 expression of BAT in IR^FKO^-denervated mice. Our data showed that BAT denervation significantly reduces LPD-induced FGF21 expression in BAT and circulating FGF21 levels (Figs. 5D & 5E). Taken together, our current data suggests that FGF21 potentially mediates the LPD-induced blood glucose normalization in the IR^FKO^ mice.

## Discussion

In the past decades, the alarming rise in the prevalence of metabolic diseases, including insulin resistance and diabetes, has invigorated interest in developing complementary therapeutic strategies. Reducing dietary proteins has recently been shown to promote or preserve metabolic health in young mice and rats^29,30,33^ and obesity-induced metabolic dysfunction rodent models^34–36^. This study tested the therapeutic potential of protein restriction (LPD) on a diabetic lipodystrophic mouse model (adipose tissue-specific insulin receptor knockout, IR^FKO^)^22^. The IR^FKO^ mice display lipodystrophy associated with white and brown adipose tissue dysfunction, hepatosteatosis, and profound insulin resistance characterized by severe hyperglycemia. These phenotypes are consistent with other published pre-clinical models of lipodystrophy-associated diabetes and lipodystrophy diabetes in human patients^37–39^, making it a unique model for studying lipodystrophy diabetes. Moreover, since insulin signaling plays critical roles in various metabolic processes, including glucose uptake, lipolysis, and lipogenesis in adipocyte^40–42^, our model will also directly test the requirement of adipocyte insulin signaling in the LPD-induced beneficial metabolic effects.

Most studies conducted so far have demonstrated beneficial metabolic effects at least after 4 weeks of LPD treatment^15,35,43,44^. Unexpectedly, we observed a significant reduction in blood glucose as early as 10 days after initiating LPD treatment. By the end of 2 weeks of treatment, protein restriction normalized IR^FKO^ mice hyperglycemia, reduced plasma insulin, improved glucose tolerance, and enhanced insulin sensitivity of IR^FKO^ mice to WT levels. These parameters also improved in WT mice after 2 weeks of short-term protein restriction, consistent with previous studies^34^. However, we did not observe glycemic variation in WT mice, likely due to intact mechanisms of glucose homeostasis in this genotype. Simultaneously, LPD decreased the body weight of IR^FKO^ and WT mice, consistent with previous studies^16,21,29,34,35^. Since LPD reduces body weight in both genotypes and decreases glycemia only in IR^FKO^ mice, we infer that the beneficial effects of LPD on glucose homeostasis are independent of body weight reduction in the IR^FKO^ model.

Based on protein leverage hypothesis^45–47^, animals are expected to increase food consumption to compensate for the protein requirement. However, we did not observe altered food intake in the LPD experimental groups, which is consistent with recent studies that showed even protein levels of 1 and 2.5% did not induce hyperphagia^17,34,48^. Based on their data, they concluded that energy intake is linked to dietary fat and not protein or sucrose in C57BL/6 mice^34^. Nevertheless, it is important to note that other studies in mice and rats^29,48^ did observe a small increment in energy intake in the animals which are protein restricted. Differences in energy intake could be due to the length of dietary treatment, the nutritional status of the experimental model before the treatment (fasted, calorie-restricted, ad-libitum fed, type of diet fed), macro and oligo-nutrient ratios of the diet, and accuracy of energy intake measurements. More accurate feeding behavior experiments are required to clarify this discrepancy.

Interestingly, WT and IR^FKO^ mice showed increased EE the day after we introduced LPD with no energy intake or movement changes. While previous studies have also indicated an increment for EE with a 5% protein diet, this effect was observed after 3 days of treatment^16^. This discrepancy could be due to the differences in the intrinsic characteristics of the chosen indirect calorimetry system. Current commercial available indirect calorimetry systems have been shown to differ in their assessments of EE, food intake, and RER in mice, presumably due to differential sensitivities of the systems (resolution of endpoint detection, endpoint detection limits, or time-resolution between measurements, etc.), effects of system design upon the cage microenvironment (floor type, air flow rate, noise levels and types, food hopper designs, environmental enrichment, etc.), and study design (diets supplied, ambient temperature, mouse strains, etc.)^49^. Nevertheless, our indirect calorimetry results suggest that the decreased body weight induced by LPD feeding is primarily due to increased EE but not reduced food intake. In line with this hypothesis and other previous reports^50,51^, LPD feeding in WT and IR^FKO^ mice strongly actives the BAT thermogenesis, a critical contributor to adaptive energy expenditure^52–54^.

It is worth noticing that the contribution of BAT to the LPD-induced beneficial metabolic effects could be exaggerated in IR^FKO^ mice compared to WT mice. Due to their lipodystrophy, IR^FKO^ mice have extremely low levels of subcutaneous white adipose tissue, which also significantly contribute to LPD-induced increases in energy expenditure through browning^55^. Based on these observations, we set out to study if sympathetic activation of BAT is required for the positive effects of LPD in IR^KFO^ mice. It is important to note that besides thermogenesis, BAT also plays a critical role as a metabolic sink for glucose, lipid, and branch-chained amino acids (BCAA)^14,56−59^. Consistently, BAT surgical denervation attenuated LPD-induced metabolic changes, including the ‘browning’ effects on BAT, energy expenditure-promoting response, and the glucose-ameliorating results, but not the body weight-lowering effects. These results suggest that sympathetic BAT activation induced by LPD positively affects both energy and glucose homeostasis in IR^KFO^ mice. Considering the genetic nature of the IR^KFO^ model, the LPD-induced BAT glucose uptake is at least partially mediated by an insulin-independent mechanism, different from the previous view that LPD lowers blood glucose primarily through enhancing BAT insulin sensitivity^60,61^. This is in line with a previous study that has identified a novel sympathetic/β3-adrenoceptor/mTORmediated iBAT glucose uptake, independent of the classical insulin/phosphoinositide 3-kinase/Akt pathway^62^.

Previous studies have linked the beneficial metabolic effects of dietary protein restriction to FGF21, a starvation-induced peptide hormone secreted mainly by the liver^63^. Interestingly, we observed that chow-fed IR^FKO^ mice have basal plasma FGF21 100 times higher than chow-fed WT mice, presumably due to the intrinsic severe lipodystrophy and diabetes in IR^FKO^ mice. In supporting this point of view, previous reports have shown significantly increased plasma FGF21 in lipodystrophy mice and humans as well as diabetic patients^64,65^. Furthermore, FGF21 plasma levels have been proposed as a marker for the following lipodystrophy in human immunodeficiency virus (HIV)-positive patients^66^.

Notably, we found that FGF21 mRNA gene expression in the liver and BAT, as well as FGF21 plasma levels, are significantly upregulated after LPD feeding in both WT and IR^FKO^ mice. Interestingly, in IR^FKO^ mice, blunted-sympathetic input to BAT through denervation effectively reduced BAT expression and plasma levels of FGF21, suggesting that intact sympathetic stimulation is necessary for IR^FKO^ BAT to produce FGF21 in response to protein restriction. Considering the essential role of FGF21 in glucose utilization, lipid metabolism, and whole-body energy balance^28,44,63^, the denervation-induced drop of BAT and plasma FGF21 could potentially contribute to the attenuated beneficial metabolic effects induced by LPD feeding.

Another interesting observation is that surgical denervation only partially reduced FGF21 gene expression compared to sham mice. This result indicates that protein restriction can partly stimulate the BAT FGF21 expression gene independently of insulin signaling and sympathetic stimulation. This could be explained by intrinsic amino acid sensing of BAT, including mTOR, GCN2, AMPK, ATF4 and/or endocrine signals to BAT from other tissues like the liver^33,67−69^.

Consistent with previous reports in metabolic syndromes induced by aging^28,44^, our results discussed here focusing on a lipodystrophy diabetes mouse model consistently showed the beneficial metabolic effects of protein restriction on glucose balance and energy homeostasis.

Taken together, our data suggests that protein restriction could be a potential therapeutic strategy for the treatment of severe metabolic syndromes.

## Methods

### CONTACT FOR REAGENT AND RESOURCE SHARING

Further information and requests for resources and reagents should be directed to and will be fulfilled by the Lead Contact, Chong Wee Liew (cwliew@uic.edu)

#### Experimental Model And Subject Details

##### Animals

Adipoq-Cre (#028030) and IR^fl/fl^ (#006955) mice were initially obtained from the Jackson Laboratory. Both lines are on a C57BL/6 background. IR^fl/fl^ mice were crossed with Adipoq-Cre to generate a fatspecific insulin receptor knockout (IR^FKO^) mouse model. IR^flox/flox^ mice containing the Adipo-Cre allele were bred with IR^flox/flox^ littermates lacking the Adipo-Cre allele to generate the mice for the IR^FKO^ experiments. IR^flox/flox^ mice with the Adipoq-Cre allele were termed IR^FKO^ mice while littermates lacking the Cre allele were used as control mice in IR^FKO^ experiments. Mice were housed in environmentally controlled conditions with a 12-h light/dark cycle and had free access to standard rodent pellet food and water. The animal protocols were approved by the Institutional Animal Care and Use Committee (IACUC) of University of Illinois at Chicago. Animal care was given in accordance with institutional UIC and ARRIVE guidelines. 6–8 week-old mice were used for all the experiments. Body weights and glycemia were measured every 3 days from the start of treatment until tissue collection.

##### Diets

Control mice were fed chow diet (17% fat, 25% protein and 58% carbohydrate by kcal; #7012, Envigo, Indianapolis, Indiana, USA). Low protein-amino acid defined diet (TD. 140918) was designed for a reduction of total protein content to 5% (kcal/from). Complete macronutrient composition: kcal from protein 5%, carbohydrates 76.4% and fat 18.5%. Caloric density: 3.9 kcal/g. Diet was color coded orange by manufacturer. Detailed information on diet composition can be seen on Table 1 for chow diet and Table 2 for LPD.

##### Surgical Denervation of Interscapular BAT (BAT)

Surgical BAT denervation was performed as previously described^70,71^. Eight-week-old mice were used for this experiment. On the day of surgery, each mouse was weighed and anesthetized with isoflurane. The mouse was shaved and secured on a warm surgical table. Following a standard skin disinfection procedure with ethanol and iodine swabs, a lateral incision was made to expose the interscapular fat pads. On both sides, all five branches of intercostal sympathetic nerves connecting to the right and left BAT fat pads were identified, carefully isolated, and sectioned. After denervation, the interscapular fat pads were returned to their original positions. Mice were allowed to recover for 7 days post-surgery before being exposed to the experimental conditions.

#### In vivo glucose uptake assay

Glucose uptake assay protocol was adapted from C. Ronald Kahn’s group (Joslin Diabetes Center) method^72^. In short, overnight fasted mice were anesthetized using Tribromoethanol (Avertin) 20 minutes prior to tail vein injection of glucose tracer (14C deoxy glucose, 0.1 uCi/g, PerkinElmer). Blood glucose was measured using an automated glucose monitor (Glucometer Elite, Bayer, Bayer AG, Leverkusen, Germany) 20 minutes before tracer injection and again at 5,15,30,45 minutes to control efficiency of tail vein injections. After 45 minutes, iBAT was collected, homogenized in PBS and centrifuged. Then, 200 ul of crude lysate supernatant were incubated with either 600 ul 4,5% perchloric Acid (PCA extract) or 600 ul 3N Barium oxide + 0.3N Zinc sulfate (Ba(OH)2 extract) for 15 minutes before centrifugation. Finally, 550 ul of supernatants were taken to radioactivity counting on Beckman counter (LS-6500) with 6 ml of Scinti-fluid (PerkinElmer). Final glucose uptake was calculated from counts per minutes (cpm)/per gram of tissue of PCA extracts minus Ba(OH)2 extracts.

##### Food intake, energy expenditure, physical activity, and body composition

Food intake, oxygen consumption, carbon dioxide production, energy expenditure, and physical activity were measured using the Promethion System (Sable Systems International, Las Vegas, NV, USA). The energy expenditure was normalized to lean body mass. The respiratory exchange ratio (RER) was calculated using the VCO2/VO2 ratio from the gas exchange data. Body composition (lean and fat mass) was estimated using NMR (Bruker minispec LF50, Billerica, MA, USA).

##### Physiological studies and Histological Analyses

Blood glucose was measured using an automated glucose monitor (Glucometer Elite, Bayer, Bayer AG, Leverkusen, Germany). Tolerance tests were performed as described previously^73^. Briefly, mice underwent a 16h overnight or 2h morning fast, before the glucose (IPGTT) and insulin (IPITT) tolerance tests, respectively. During experiments with IR^FKO^ mice, mice were injected intraperitoneally (IP) with a bolus of 2g/kg dextrose (Hospira, Lake Forest, IL, USA) for IPGTT or 1.5U/kg humalin (Eli Lilly, Indianapolis, IN, USA) for IPITT. Plasma insulin and FGF21 were measured with an ELISA kit (Crystal Chem: #90080 and R&D systems: #MF2100 respectively). Non-esterified fatty acids (NEFA), triglyceride (TG), and cholesterol concentration in serum were measured with NEFA-C, Triglyceride E tests (Wako) and cholesterol liquicolor kits (Stanbio), respectively. For histological analyses, mice were sacrificed, and tissues were excised, weighed, and processed for immunohistochemistry as described previously^74^. Adipose tissue depots were fixed for 48h in formalin and immediately processed. Tissues were sectioned by the Human Tissue Resource Center (University of Chicago, Chicago, IL, USA).

##### RNA extraction and real time PCR

Total RNA was isolated from tissues using Trizol reagent (Invitrogen, Carlsbad, CA, USA) and the Directzol Kit (Zymo, Irvine, CA, USA). cDNA was generated from 2ug of total RNA using the High-Capacity cDNA Reverse Transcription Kit (Invitrogen, Carlsbad, CA, USA) using random hexamer primers, following the manufacturer’s instructions. The resulting cDNA was diluted to a concentration of 10ng/uL and 1.67uL was aliquoted for each 6.67uL real-time quantitative polymerase chain reaction (qPCR) (SYBR Green, BioRad, Hercules, CA, USA). Reactions contained primers (Integrated DNA Technologies, Coralville, IA, USA) at a concentration of 300nM each, and 600nM for UCP1 in white adipose tissue. PCR reactions were run in duplicate and quantitated using the ViiATM7 Real-Time PCR system (Applied Biosystems, Foster City, CA, USA). Results were normalized to TATA box binding protein (TBP) expression and expressed as arbitrary units or fold change with respect to control. Primer sequences: UCP1 Fw: CTGCCAGGACAGTACCCAAG; UCP1 Rv: TCAGCTGTTCAAAGCACACA. DIO2 Fw: AAGGCTGCCGAATGTCAACGAATG; DIO2 Rv: TGCTGGTTCAGACTCACCTTGGAA. FGF21 FW: GCTCTCTATGGATCGCCTCAC; FGF21 Rv: GGTACACATTGTAACCGTCCTC. BMP8b Fw: GTGGTCCAAGAGCACTCCAACA; BMP8b Rv: GGTCCTTGTGATGGTTCAGCAG.

#### Quantification And Statistical Analysis

Sample sizes were determined using prior characterization experiments for the mouse models used for this study. Mice were randomized for treatment in a blinded manner when possible.

All data are presented as the mean ± standard error of mean (SEM) and were analyzed by one-way ANOVA test with a threshold for significance of 0.05 adjusted P value. For blood glucose, bodyweight, IPGTT and IPITT measurements, data was analyzed using two-way ANOVA test with a threshold for significance of 0.05 adjusted P value. Energy expenditure measurements comparisons were analyzed using paired Student’s t-test of values corresponding to each mouse before and after diet change. *= P > 0.05; **=P > 0.01; ***=P > 0.001; ****=P > 0.0001.

## Figures and Tables

**Table 1:** CHOW DIET INFORMATION #7012-

Macronutrients		
Energy Density d	kcal/g (kJ/g)	3.1
Calories from Protein	%	25
Calories from Fat	%	17
Calories from Carbohydrate	%	58
Amino Acids		
Aspartic Acid	%	1.8
Glutamic Acid	%	2.8
Tyrosine	%	0.8
Methionine	%	0.4
Cystine	%	0.3
Alanine	%	1.0
Glycine	%	0.8
Threonine	%	0.8
Proline	%	1.4
Serine	%	1.3
Leucine	%	1.7
Isoleucine	%	0.8
Valine	%	0.9
Lysine	%	1.0
Histidine	%	0.5
Arginine	%	1.2
Tryptophan	%	0.3
Phenylalanine	%	0.9

**Table 2:** LOW PROTEIN DIET INFORMATION # TD.140918

Macronutrients		
Energy Density d	kcal/g (kJ/g)	3.9
Calories from Protein	%	5.1
Calories from Fat	%	18.5
Calories from Carbohydrate	%	76.4
Amino Acids (g/KG) g/Kg		
L-Alanine	2.18	
L-Arginine	1.46	
L-Asparagine	4.79	
L-Aspartic Acid	4.79	
L-Cystine	1.67	
L-Glutamic Acid	6.74	
L-Glutamine	7.87	
Glycine	0.69	
L-Histidine HCl, monohydrate	1.07	
L-Isoleucine	1.81	
L-Leucine	5.9	
L-Lysine HCl	4.74	
L-Methionine	1.56	
L-Phenylalanine	1.54	
L-Proline	1.72	
L-Serine	1.72	
L-Threonine	2.26	
L-Tryptophan	0.79	
L-Tyrosine	1.61	
L-Valine	1.95	

## Data Availability

All data generated during this study are included in this published article.
